# Decline in EBV-Specific IFN T cell responses in Kenyan infants from a malaria holoendemic region of Kenya

**DOI:** 10.1186/1750-9378-7-S1-O22

**Published:** 2012-04-19

**Authors:** Amolo S Asito, Erwan Piriou, Odada P Sumba, Ann M Moormann, Rosemary Rochford

**Affiliations:** 1 SUNY Upstate Medical University, Syracuse, NY, USA; 2 Center for Global Health Research, Kenya Medical Research Institute, Kisumu, Kenya; 3 University of Massachusetts Medical School, Worcester, MA, USA

## Background

Endemic Burkitt’s lymphoma, the most prevalent childhood cancer in Equatorial Africa, is a rapidly growing B-cell malignancy that is ultimately fatal if untreated. Two co-factors are linked to the etiology of this pediatric cancer: Epstein-Barr virus (EBV) infection, and sustained and intense exposure to *Plasmodium falciparum* malaria (holoendemic malaria). In this study, we wanted to test the hypothesis that *P. falciparum* infections during early infancy results in elevated EBV viral load which results in diminished EBV-specific T-cell immune responses over time.

## Methods

Infants were enrolled from two rural sites in Kenya: Kisumu District where malaria transmission is holoendemic and risk for eBL is high and Nandi District where malaria transmission is limited and the risk for eBL is low. Finger prick blood samples were taken through 2 years of age to measure EBV viral load, EBV antibodies, and malaria parasitemia. Venous blood samples were collected at 12, 18 and 24 months of age and PBMC were isolated and stimulated with peptides for both EBV lytic and latent antigens. After 2.5 days of stimulation, IFNγ ELISPOTS enumerated EBV-specific T cell responses, and the number of SFU/10^6^ PBMC was determined by scanning with ImmunoSpot Reader and Software.

## Results

When we compared EBV lytic and latent IFNγ T cell responses at 12, 18 and 24 months of age, we saw that although children in Kisumu were able to mount an IFNγ response against EBV lytic peptides, the magnitude of that response declined significantly by 24 months of age. In contrast, the magnitude of the response did not decline in the Nandi cohort. We also observed higher overall viral loads in infants from Kisumu suggesting that the apparent loss of EBV-specific IFNγ response to lytic antigens in the Kisumu children may be associated with these higher viral loads.

## Conclusions

We found that by 2 years of age, there was a significant difference in the capacity of children living in a malaria holoendemic region compared to malaria sporadic region to maintain a T cell response to EBV lytic antigens. This suggests that *P. falciparum* malaria contributes to loss of EBV-specific immunity by inducing the collapse of an antiviral IFN-γ mediated CD8+ T cell response.

